# Chrononutrition and Diabetes: Mapping the Scientific Landscape Through Bibliometric Insights

**DOI:** 10.7759/cureus.93538

**Published:** 2025-09-30

**Authors:** Sushil Kumar, Abha Kumari, Pradosh Kumar Sarangi, Sudip Bhattacharya, Alok Singh, Akanksha Singh, Seshadri Reddy Varikasuvu, Himel Mondal

**Affiliations:** 1 General Medicine, All India Institute of Medical Sciences, Deoghar, Deoghar, IND; 2 Community and Family Medicine, All India Institute of Medical Sciences, Deoghar, Deoghar, IND; 3 Radiodiagnosis, All India Institute of Medical Sciences, Deoghar, Deoghar, IND; 4 Community Medicine, Shree Guru Gobind Singh Tricentenary University, Gurugram, IND; 5 Ayurvedic Medicine, Mahatma Gandhi Kashi Vidyapith, Varanasi, IND; 6 Biochemistry, All India Institute of Medical Sciences, Deoghar, Deoghar, IND; 7 Physiology, All India Institute of Medical Sciences, Deoghar, Deoghar, IND

**Keywords:** chrononutrition, circadian rhythm, diabetes, dietary interventions, glycemic control, insulin resistance, intermittent fasting, meal timing, metabolic health, time-restricted feeding

## Abstract

Chrononutrition, an emerging field at the interface of circadian biology, nutrition, and metabolic health, has gained increasing attention in the context of diabetes research. To capture the evolution and current state of this domain, we conducted a bibliometric analysis of publications indexed in the Scopus database until August 31, 2025. The dataset comprised 1,629 documents retrieved across 678 sources, which were analyzed using Biblioshiny and VOSviewer. The analysis revealed an annual growth rate of 11.41%, reflecting steady and accelerating global interest in the role of meal timing and circadian rhythms in diabetes. The United States, China, and Australia emerged as the most productive countries, while the University of Illinois at Chicago, Adelaide Medical School, and Universiteit van Amsterdam ranked as leading institutions. International collaboration was extensive, with the United States acting as a central hub connecting research networks across continents. Citation trends showed increasing influence of the field after 2000, peaking between 2010 and 2018, followed by a relative decline in recent years. Thematic mapping highlighted the following three major clusters (keyword co-occurrence): clinical trials on glycemic control and lifestyle, experimental animal studies on circadian biology, and dietary interventions such as time-restricted feeding and intermittent fasting. This study underscores the multidisciplinary growth of chrononutrition in diabetes research, highlights its translational potential for clinical practice, and identifies opportunities for broader global participation and culturally diverse interventions.

## Introduction and background

Diabetes mellitus has emerged as one of the most significant global health challenges, with its prevalence continuing to rise at an alarming rate [1]. The prevention and management of diabetes largely depend on lifestyle modifications, among which diet plays a central role. In recent years, attention has shifted beyond the composition of diet to its temporal aspects, giving rise to the concept of chrononutrition [2]. Chrononutrition explores the interaction between the timing of food intake and the body’s circadian system, emphasizing that not only what we eat but also when we eat can influence metabolic outcomes [3].

Mounting evidence suggests that irregular eating patterns, late-night meals, and circadian misalignment contribute to impaired glucose metabolism and increased risk of type 2 diabetes [4]. Conversely, dietary strategies such as time-restricted feeding, intermittent fasting, and alignment of meal timing with circadian rhythms have shown promise in improving insulin sensitivity and glycemic control [5]. As these insights continue to accumulate, chrononutrition has become an evolving research frontier with implications for the prevention and management of diabetes [6].

Given the rapid expansion of literature in this interdisciplinary field, a structured mapping of scientific output is needed to capture its evolution, key contributors, collaborative networks, and emerging themes. Bibliometric analysis provides a robust methodological approach to evaluate the growth, impact, and intellectual structure of research [7]. By applying bibliometric techniques to the literature on chrononutrition and diabetes, it is possible to identify research hotspots, trace thematic developments, and highlight influential authors, institutions, and countries driving this domain forward.

The present study aims to conduct a bibliometric analysis of global research on chrononutrition and diabetes, with the objective of characterizing publication trends, research collaborations, and thematic evolution within this rapidly advancing field. Given the growing recognition of chrononutrition as a determinant in diabetes management, a systematic mapping of the literature is essential to consolidate existing knowledge. To our knowledge, this is the first bibliometric study that integrates publication trends, collaboration networks, and thematic evolution in this emerging research domain.

## Review

Methodology

Data Source and Search Strategy

The bibliometric data for this study were retrieved from the Scopus database (Elsevier, Amsterdam, the Netherlands), one of the largest multidisciplinary repositories of peer-reviewed literature. The search was conducted on August 31, 2025, without restrictions on publication year, to capture the full scope of research output on the topic. No language filter was applied.

The search strategy combined terms related to chrononutrition and diabetes. Specifically, chrononutrition-related terms included “chrono-nutrition,” “chrononutrition,” “meal timing,” “time-restricted feeding,” “intermittent fasting,” “eating window,” “circadian eating,” “meal frequency,” “diet timing,” and “feeding time.” These were cross-referenced with diabetes-related terms, namely “diabetes,” “type 2 diabetes,” “T2DM,” “type 1 diabetes,” “insulin resistance,” “prediabetes,” “glucose metabolism,” and “glycemic control.”

The resulting records were exported in CSV format, with full citation information, abstracts, keywords, and cited references for subsequent analysis.

Inclusion and Exclusion Criteria

The inclusion criteria for this bibliometric study encompassed all documents indexed in the Scopus database up to the search date of August 31, 2025, without restriction on publication year or language. Documents were included if they addressed both chrononutrition and diabetes in their title, abstract, or body text. The study considered peer-reviewed articles, including original research articles, reviews, conference papers, and book chapters, as available in the Scopus database. No exclusion criteria were selected in terms of language, date, country, or type of study.

Data Processing and Bibliometric Analysis

The dataset was analyzed using Biblioshiny, a web-based application of the Bibliometrix R-package (developed by Massimo Aria and Corrado Cuccurullo, University of Naples Federico II, Naples, Italy) [8]. Biblioshiny was employed to generate descriptive statistics; evaluate publication trends; identify prolific authors, institutions, and countries; and map thematic evolution.

For visualization and network analysis, VOSviewer (developed by Nees Jan van Eck and Ludo Waltman, Centre for Science and Technology Studies, Leiden University, Leiden, the Netherlands) was utilized [9]. VOSviewer enabled the construction of co-authorship, keyword co-occurrence, and citation networks, highlighting patterns of collaboration and research hotspots.

Ethical Considerations

As this study was based exclusively on bibliometric data obtained from the Scopus database and did not involve human participants, patient data, or animal experiments, ethical approval was not required.

Results

**Figure 1:**
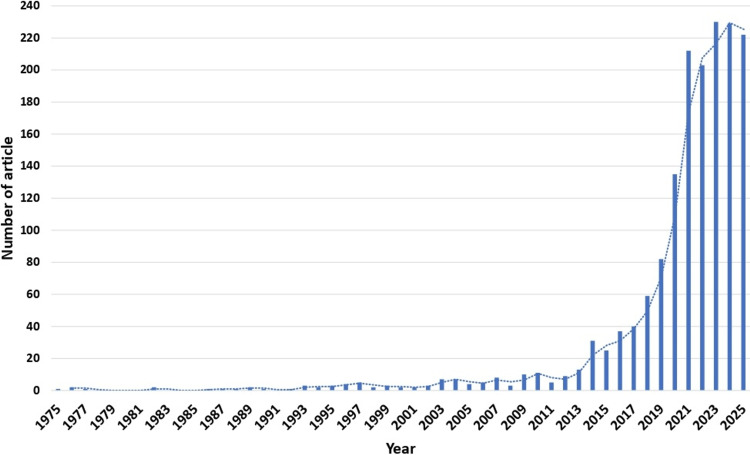
Year-wise publications on chrononutrition and diabetes (data up to August 31, 2025).

The bibliometric analysis retrieved a total of 1,629 documents on chrononutrition and diabetes, published between 1975 and 2025, across 678 different sources. Research in this field demonstrated a strong upward trajectory with an annual growth rate of 11.41%, reflecting increasing global attention. Year-wise publication is shown in Figure 1.

**Figure 2:**
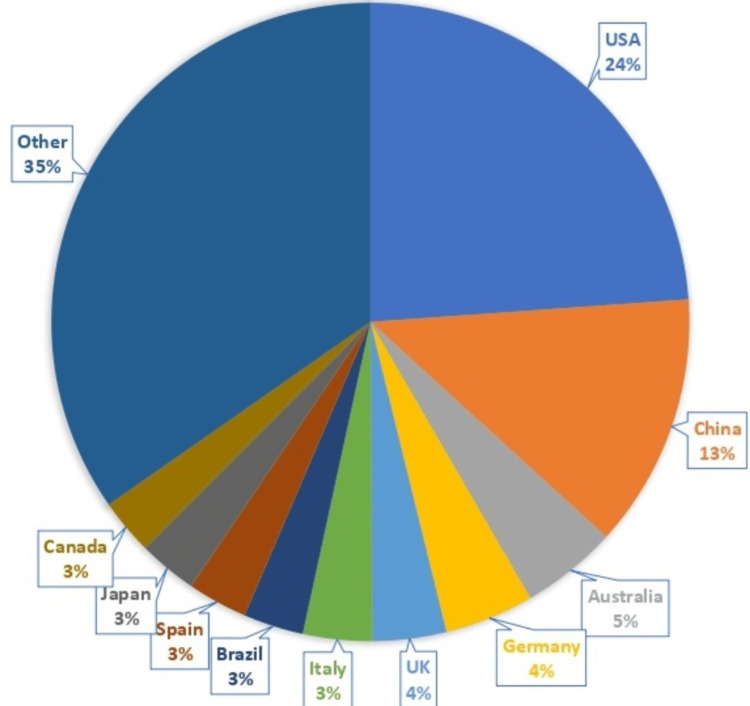
Number of publications according to country.

The analysis of country-level contributions revealed that the United States (number of authors affiliated (n) = 2,037), China (n = 1,099), and Australia (n = 417) were the leading contributors to publications on chrononutrition and diabetes. Other major contributors included Germany, the United Kingdom, Italy, Brazil, Spain, Japan, and Canada, along with a wide range of additional countries. These global patterns of research productivity are illustrated in Figure 2.

**Figure 3:**
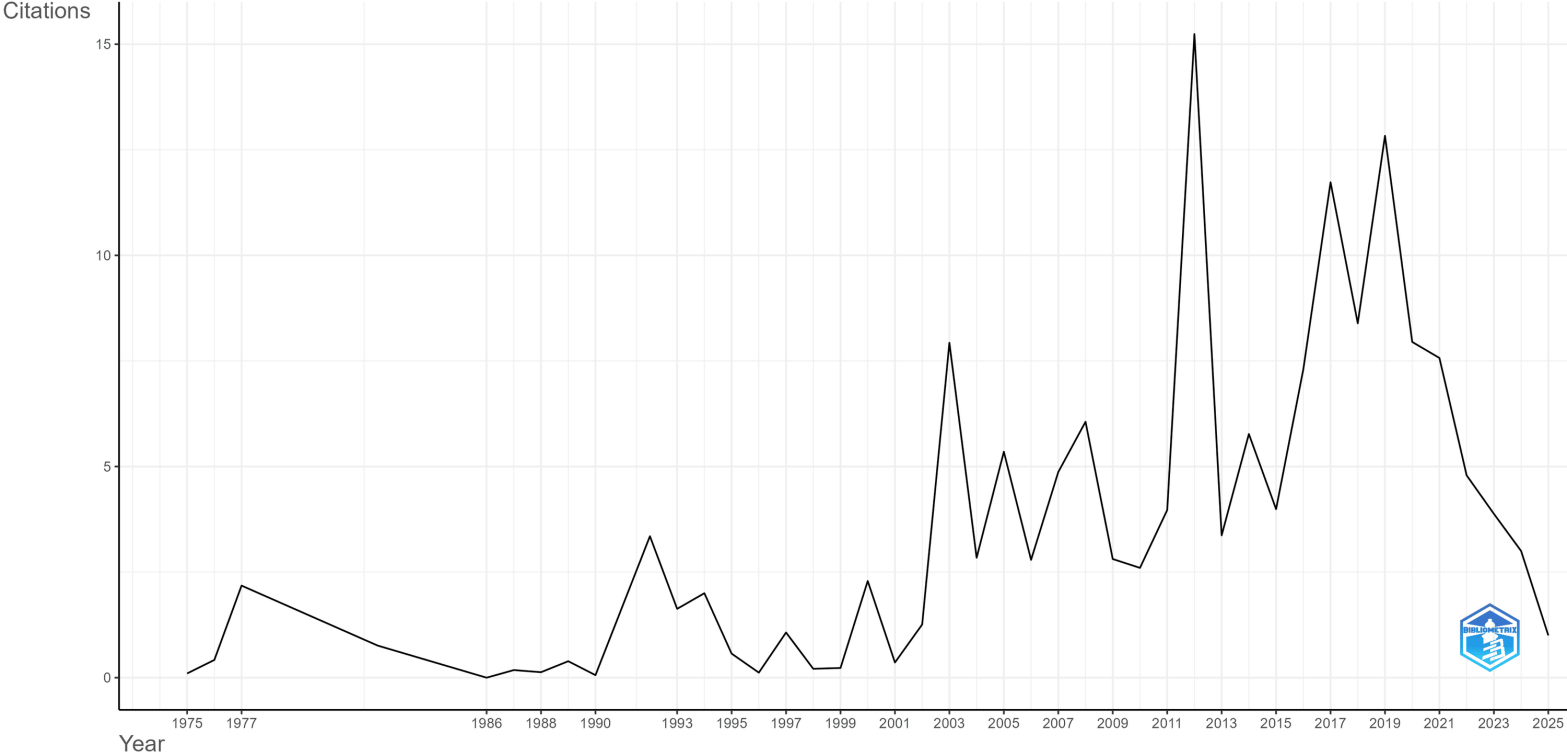
Trend of average citations per year in the field of chrononutrition and diabetes (data up to August 31, 2025).

The trend of average citations per year in the field of chrononutrition and diabetes demonstrated distinct fluctuations across the study period (1975-2025). In the early years (1975-1995), publications received relatively few citations, reflecting the nascency of the field and limited scholarly attention. A gradual rise was observed after 2000, coinciding with increasing interest in circadian biology and nutrition science. Between 2010 and 2018, the field reached its peak citation impact, with certain years averaging more than 15 citations per article, highlighting a surge in influential research outputs and heightened global attention. However, from 2019 onwards, a steady decline in average citations per year was noted (Figure 3).

**Figure 4:**
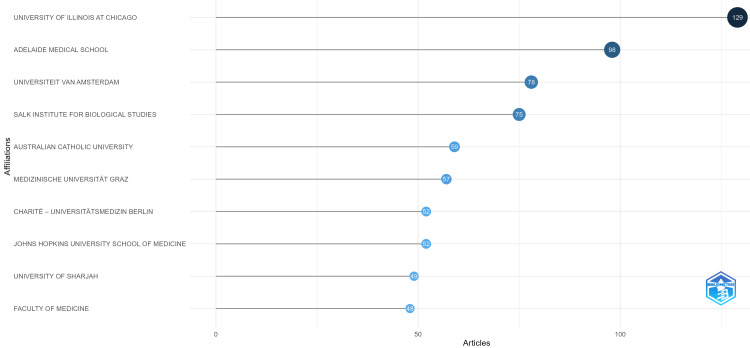
Top institutions with their number of publications.

Figure 4 shows the research productivity in chrononutrition and diabetes. The University of Illinois at Chicago leads as the most productive institution with 129 published articles, followed by Adelaide Medical School, which ranks second with 98 articles, and Universiteit van Amsterdam, which takes third place with 78 articles.

**Figure 5:**
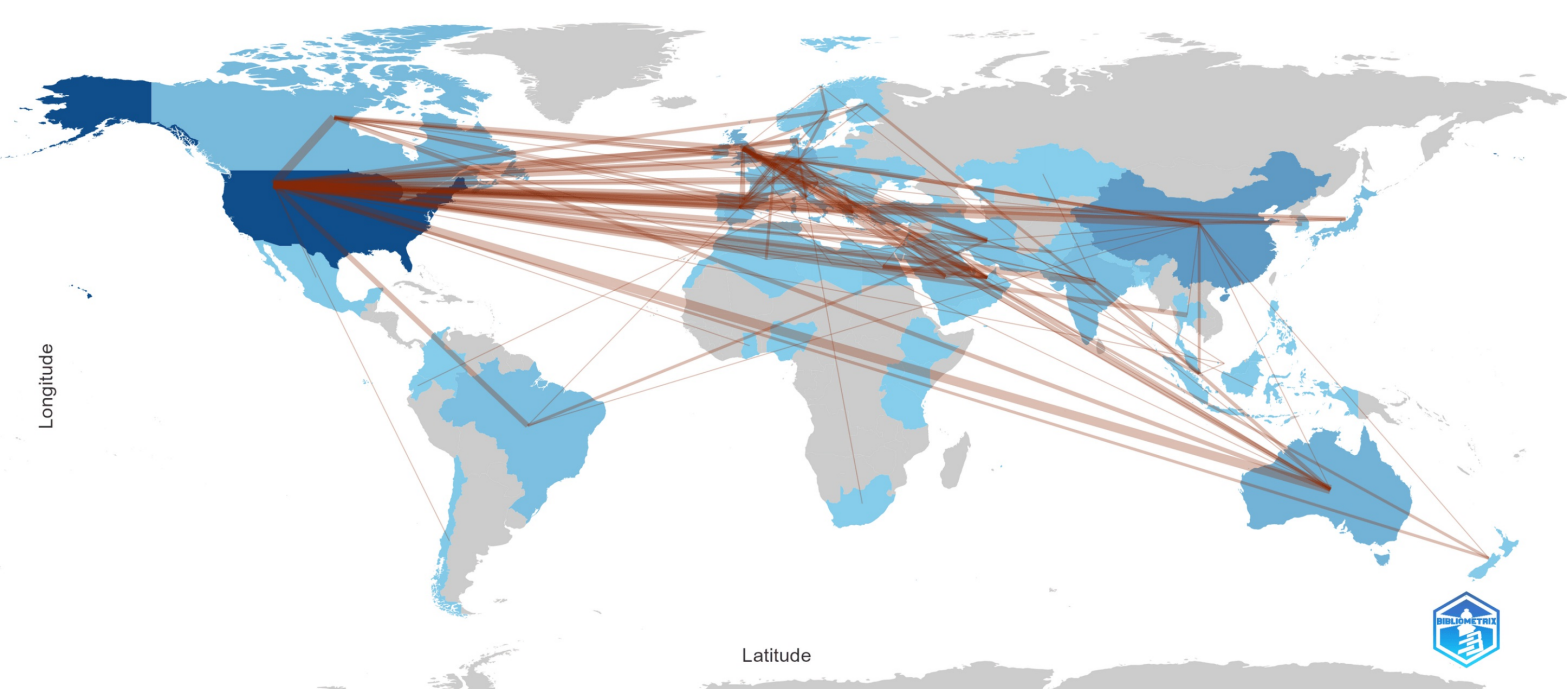
Country collaboration map showing the collaboration among countries connected by brown lines. Blue intensity shows the number of publications per country (darker = more). Brown lines represent international collaborations (thicker = stronger).

The country collaboration map (Figure 5) reveals extensive international research partnerships in chrononutrition and diabetes studies, with the United States serving as a central hub connecting to multiple continents through dense collaboration networks. Europe demonstrates strong intra-regional cooperation, particularly among countries like the UK, Germany, the Netherlands, and other European Union nations, while also maintaining robust transatlantic partnerships with North America. Australia and China emerge as significant Asia-Pacific collaboration centers, with Australia showing extensive connections to both European and American institutions, and China displaying growing research partnerships across global networks, highlighting the truly international nature of chrononutrition and diabetes research efforts.

**Figure 6:**
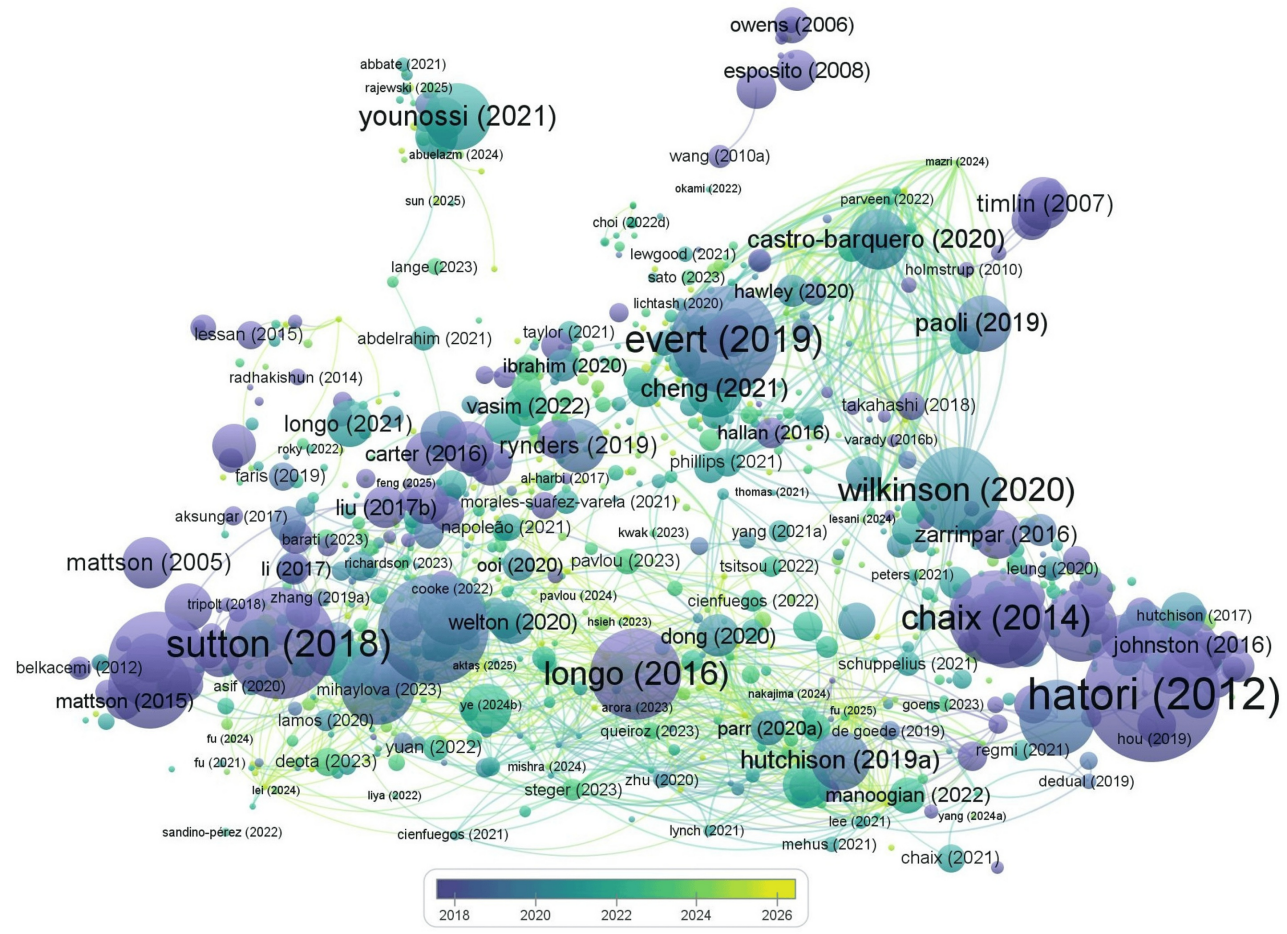
Bibliographic coupling map highlighting influential and interconnected publications in chrononutrition and diabetes research.

A total of 11,369 authors contributed to the literature, with an average of 16.1 co-authors per document, underscoring the collaborative nature of this research domain. Notably, there were no single-authored documents, indicating that the field is primarily driven by multi-author collaborations. International research collaboration was also significant, with 22.41% of publications involving co-authorship across countries. The bibliographic coupling map highlights the most influential and interconnected publications in the field of chrononutrition and diabetes research (Figure 6). Key nodes with the strongest coupling include Sutton (2018), Hatori (2012), and Evert (2019), which represent landmark works shaping the research landscape. Other highly connected publications, such as Wilkinson (2020), Longo (2016, 2021), and Chaix (2014), also demonstrate strong relevance and frequent co-citation, suggesting their foundational role in guiding subsequent studies. The dense clustering and interlinking between more recent works (e.g., Younossi 2021, Paoli 2019, Castro-Barquero 2020) and earlier influential studies indicate an evolving but cohesive research network. Overall, the map underscores both continuity and expansion in the field, where earlier seminal works continue to influence emerging research directions.

**Figure 7:**
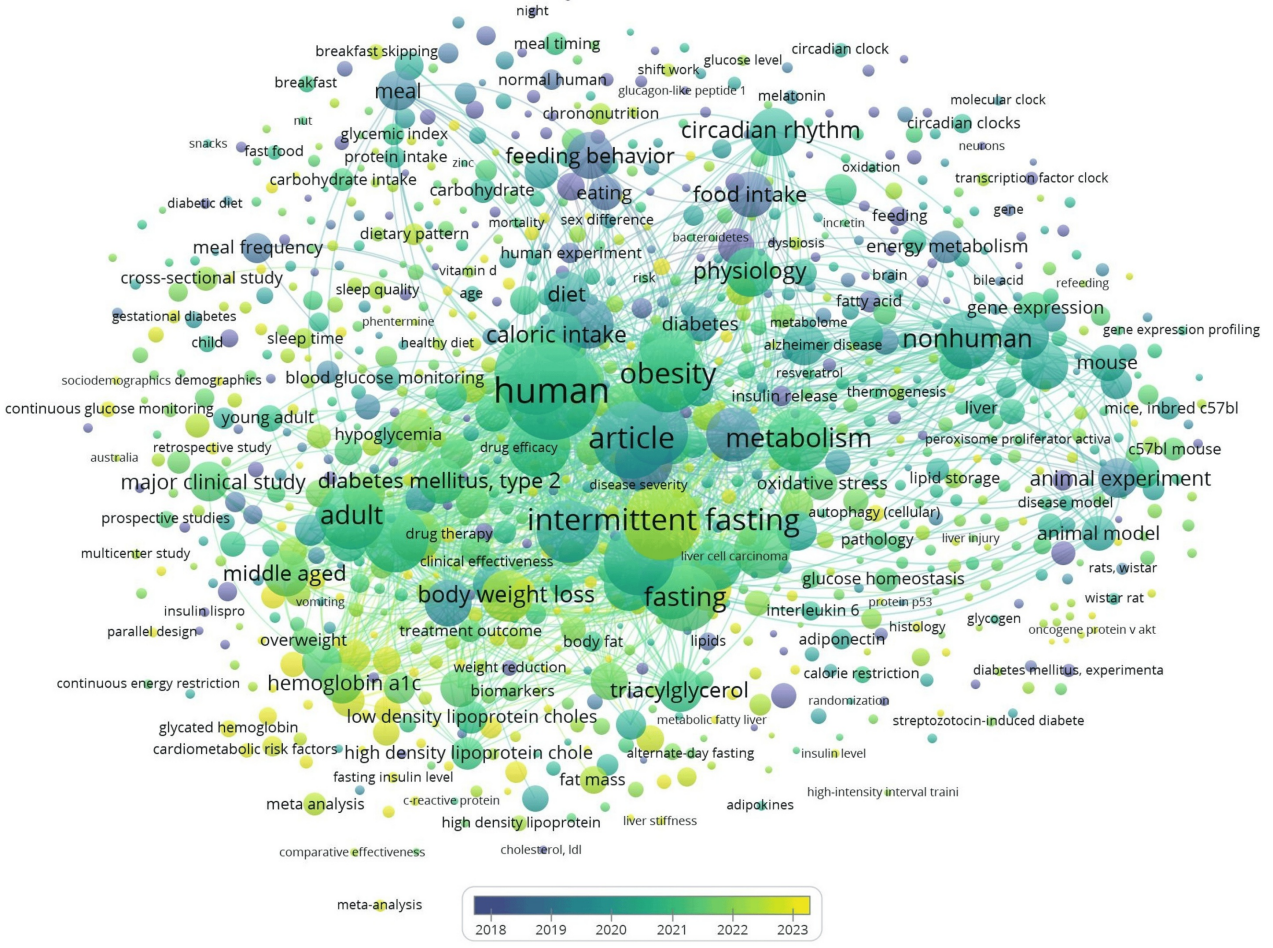
Network visualization of keyword cooccurrence.

The keyword co-occurrence network, as shown in Figure 7, reveals that chrononutrition and diabetes research centers around three major thematic clusters, namely, circadian rhythm and molecular clock mechanisms, metabolic processes and clinical outcomes, and dietary interventions and physiological responses. The visualization shows strong interconnections between human studies and animal models, indicating that research progresses from basic mechanistic studies in laboratory animals to clinical applications in humans. The temporal color gradient from 2018 to 2023 demonstrates the field’s evolution toward more integrated approaches linking circadian biology, nutritional interventions, and metabolic health outcomes.

**Figure 8:**
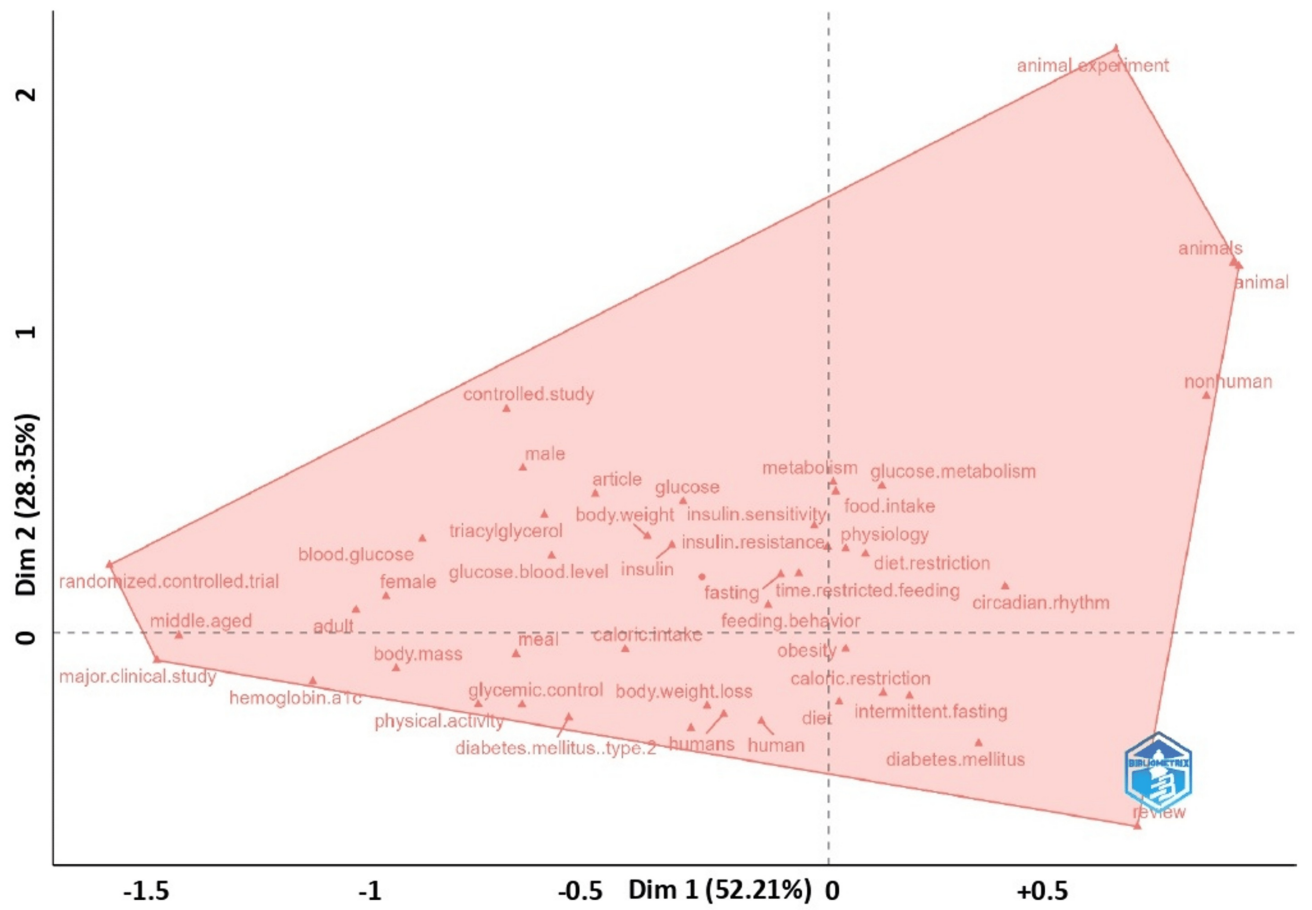
Factorial map shows the main thematic clusters of the field. The dimensions (Dim 1 and Dim 2) are statistical constructs that reduce the complexity of keyword co-occurrence patterns into two main axes, allowing clusters of related terms to be visualized in two-dimensional space. The percentage values indicate how much of the total conceptual variance is captured by each dimension.

The conceptual structure map highlighted three interconnected research domains in chrononutrition and diabetes. One cluster focused on clinical studies, emphasizing randomized controlled trials, hemoglobin A1c, body mass, and physical activity (Figure 8). A second cluster represented experimental research, with strong contributions from animal studies exploring metabolism, glucose regulation, and circadian biology. The third cluster centered on lifestyle and dietary interventions, including time-restricted feeding, intermittent fasting, caloric restriction, obesity, and glycemic control. Together, these clusters reflect a multidisciplinary field bridging mechanistic insights, lifestyle strategies, and clinical applications for diabetes management.

Discussion

This bibliometric analysis provides a comprehensive overview of the scientific landscape in chrononutrition and diabetes research, highlighting its evolution, collaborative nature, and emerging directions. The steady growth of publications underscores that this domain has shifted from a niche interest to a globally recognized field, reflecting broader acknowledgment of the role of circadian rhythms and meal timing in metabolic health [10]. The concentration of research in leading countries and institutions indicates strong hubs of expertise, but also suggests an uneven distribution of knowledge production, which could limit the representation of region-specific dietary and cultural contexts.

The findings emphasize the multidimensional nature of chrononutrition research, bridging experimental animal studies, mechanistic insights, and clinical applications [11]. The convergence of basic science with clinical and lifestyle interventions points toward a translational trajectory, where discoveries in circadian biology are increasingly applied to improve diabetes prevention and management [6]. This integration has important implications for clinical practice, as it suggests that beyond dietary composition, the timing and frequency of meals may play a pivotal role in glycemic control and long-term metabolic outcomes [12,13].

Looking forward, the field would benefit from larger, culturally diverse clinical trials on chrononutrition to validate and generalize findings across populations [14]. Strengthening collaborations between basic scientists, clinicians, and public health experts is essential to move from mechanistic understanding toward real-world applications. According to a 2023 National Heart, Lung, and Blood Institute Workshop, advancing the emerging field of chrononutrition will require several key steps, including the standardization of terminology and metrics, the development of scalable and precise tools that can be applied in real-world settings, and careful consideration of individual differences that may serve as effect modifiers. Equally important is a deeper understanding of the social, behavioral, and cultural factors that influence dietary patterns and their interaction with circadian biology [15].

Recent investigations have reinforced the importance of chrononutrition in diabetes management. Randomized controlled trials demonstrated that early time-restricted eating, particularly with carbohydrate intake, improved glycaemic control, insulin response, and weight loss in individuals with type 2 diabetes, sometimes more effectively than conventional calorie counting [16-18]. Evidence links breakfast skipping and late-night eating with circadian misalignment, obesity, insulin resistance, and type 2 diabetes, underscoring the need to optimize eating schedules for disease prevention and control [19]. However, current evidence is limited by small sample sizes, short trial durations, and a lack of validated tools to capture temporal eating patterns in diverse populations [20].

Despite these contributions, the study has limitations inherent to bibliometric analyses. Reliance on a single database (Scopus) may have excluded relevant publications indexed elsewhere, potentially introducing database bias. However, it was not possible to merge multiple databases for an analysis in Bibioshiny. Citation-based metrics may also underrepresent newer but impactful studies that have not yet accumulated citations. Furthermore, bibliometric mapping captures structural and thematic patterns but does not assess the quality or strength of scientific evidence. This study did not include an analysis of bibliographic coupling among journals or an assessment of the key sponsors supporting research in this domain. Incorporating such analyses in future work could provide deeper insights into which journals, publishers, and funding bodies have played more prominent roles in shaping scholarship in this area.

## Conclusions

The bibliometric analysis of 1,629 publications revealed rapid growth in chrononutrition and diabetes research, with strong global contributions led by the United States, China, and Australia. Collaborative, multi-author efforts dominated the field, supported by extensive international partnerships and influential landmark studies shaping its trajectory. Thematic clusters highlighted a multidisciplinary focus spanning circadian biology, clinical outcomes, and dietary interventions, underscoring the field’s evolution toward integrated strategies for diabetes management. The analysis underscores the growing importance of chrononutrition as a research frontier in diabetes, with implications for advancing both prevention and management strategies. The field is evolving rapidly, yet its future progress will depend on fostering inclusive collaborations, addressing geographic imbalances, and ensuring the translation of experimental insights into clinical and public health interventions.
